# Evidence of a metacognitive illusion in judgments about the effects of music on cognitive performance

**DOI:** 10.1038/s41598-023-46169-x

**Published:** 2023-10-31

**Authors:** Raoul Bell, Gesa Fee Komar, Laura Mieth, Axel Buchner

**Affiliations:** Department of Experimental Psychology, Heinrich Heine University Düsseldorf, Düsseldorf, Germany

**Keywords:** Psychology, Human behaviour

## Abstract

Two experiments serve to examine how people make metacognitive judgments about the effects of task-irrelevant sounds on cognitive performance. According to the direct-access account, people have direct access to the processes causing auditory distraction. According to the processing-fluency account, people rely on the feeling of processing fluency to make heuristic metacognitive judgments about the distracting effects of sounds. To manipulate the processing fluency of simple piano melodies and segments of Mozart’s sonata K. 448, the audio files of the music were either left in their original forward direction or reversed. The results favor the processing-fluency account over the direct-access account: Even though, objectively, forward and backward music had the same distracting effect on serial recall, stimulus-specific prospective metacognitive judgments showed that participants incorrectly predicted only backward music but not forward music to be distracting. The difference between forward and backward music was reduced but not eliminated in global retrospective metacognitive judgments that participants provided after having experienced the distracting effect of the music first-hand. The results thus provide evidence of a metacognitive illusion in people’s judgments about the effects of music on cognitive performance.

## Introduction

Technological advancement gives people great control over their working and learning environments. This includes the auditory environment. For instance, music streaming services offer music playlists that supposedly help their users to concentrate during work and study. An important question is whether people are willing to, or should, make use of such offers. Driven by such questions, the focus of research on auditory distraction is shifting from how passive exposure to sounds affects working and learning to how people evaluate and choose their working and learning conditions based on their metacognitive judgments. Most research has as yet focused on metacognitive judgments of learning that refer to task-relevant stimuli in the focus of attention^1–7^. However, it is also important to understand how people judge the cognitive effects of task-irrelevant stimuli. Consequently, research on the metacognition of the effects of task-irrelevant stimuli is on the rise^8–12^. Here we focus on how people arrive at metacognitive judgments about the effects of task-irrelevant sounds on cognitive performance. In applied settings, people’s metacognitive judgments about how task-irrelevant sounds affect their performance can have important consequences. For example, people may listen to different songs to decide which song may help and which song may distract them when studying. If people’s metacognitive judgments are incorrect, they may choose to study with music that disrupts their cognitive performance, resulting in suboptimal learning outcomes. It is thus important to understand how valid these metacognitive judgments about the effects of task-irrelevant stimuli on performance are and to advance our theoretical insights into the mechanism by which people arrive at these judgments. Specifically, do people have direct access to the processes underlying auditory distraction or do they use heuristics that allow them to quickly arrive at judgments which may often be correct but carry the risk of systematic and predictable error? This is the main question that motivated the present experiments.

In a recent study on the metacognition of auditory distraction^11^, different types of metacognitive judgments were collected about the effects of task-irrelevant sounds on cognitive performance. The judgments were made with respect to the serial-recall paradigm which serves as a standard experimental paradigm for studying the effects of auditory distraction (for a review, see^13^). In this paradigm, a sequence of visually presented digits has to be recalled immediately after presentation. During the presentation of the visual target stimuli, task-irrelevant auditory distractors are played that the participants are instructed to ignore. It is well known that auditory distraction in this task is mainly determined by changes in the to-be-ignored auditory channel (e.g.^14,15^). Bell et al.^11^ have distinguished between three types of sequences that are often used as auditory distractors in laboratory studies. First, steady-state sequences consist of a single word that is repeatedly presented (e.g., *Tau Tau Tau Tau Tau Tau Tau Tau Tau*). Second, auditory-deviant sequences are identical to the steady-state sequences except that one of the repeated words in the sequence is replaced by a different word (e.g., *Tau Tau Tau Tau Tau Ohm Tau Tau Tau*). Third, changing-state sequences consist of series of different words (e.g., *Tau Ohm Gel Alm Elch Milz Steg Zwist Jod*). Consistent with the literature (e.g.^16^), the objective sound effects (the difference between each of the distractor conditions and the quiet control condition in the mean number of digits per trial that were correctly recalled in the serial-recall task) showed that steady-state sequences were least distracting and changing-state sequences were most distracting.

In the study of Bell et al.^11^, several types of metacognitive judgments about the effects of task-irrelevant sounds on performance were assessed, following procedures used to measure metacognitive judgments of learning. First, participants were asked to provide *stimulus-specific prospective metacognitive judgments* (cf.^1^). Distractor sequences that participants later had to ignore were played to the participants one by one. The participants were asked to judge how distracting or helpful they thought the sounds were going to be relative to quiet while memorizing a sequence of digits for serial recall. The task of giving stimulus-specific prospective metacognitive judgments serves as a model task for the real-world scenario that people make judgments about the effects of sound on cognitive performance based on the immediate experience of a stimulus. For example, when choosing music for studying, students may browse through their playlists, listen to each song and then decide whether the song is going to help or hurt their performance. After participants had actually ignored the sequences while memorizing the digits for serial recall, they were asked to provide *global retrospective metacognitive judgments* (cf.^17^) about how distracting or helpful the types of sounds had been relative to quiet. The task of giving global retrospective metacognitive judgments serves as a model task for the real-world scenario that people make judgments about the effects of sound on cognitive performance based on experience. For example, after studying for half a day with a specific genre of music, a student may no longer feel the need to actually listen to the songs individually before deciding whether studying with this type of music is a good or bad idea—instead, the student may base their decision on their overall impression of their past experience. In the study of Bell et al.^11^, both the stimulus-specific prospective as well as the global retrospective metacognitive judgments correctly captured the objective sound effects on performance. First, participants correctly judged all of the sound sequences to have an overall distracting rather than helpful effect on performance. Second, they correctly judged steady-state sequences to be least distracting and changing-state sequences to be most distracting.

Why did these judgments correctly reflect the relative strength of the distracting effects of the different types of sounds? Two competing explanations for this pattern of results were discussed by Bell et al.^11^. One explanation is that people have *direct access* to the processes underlying auditory distraction. With respect to the distracting effects of task-irrelevant sounds, salient changes in the auditory modality may attract the focus of attention. During the serial-recall task, the focus of attention would then be withdrawn from the rehearsal of to-be-remembered digits^18^. Within the embedded-processes model of working memory^19^, “the information in the focus of attention is the same information that the person is aware of” (p. 89), except in unusual circumstances such as neurological disorders. If distraction is determined by the degree to which distractors attract the focus of attention and if it is also adequate to equate the focus of attention with conscious awareness, then people may be aware of the factors that determine auditory distraction. Specifically, people may have direct access to the degree to which different types of sounds attract their attention.

The direct-access account thus has some prima facie plausibility for explaining why metacognitive judgments and objective sound effects were closely aligned in the study of Bell et al.^11^. However, there is an alternative explanation which is at least equally plausible. We know that metacognitive judgments in other domains are often assumed to be based on the *processing-fluency heuristic* (e.g.^3,5,20,21^). Stimuli that are fluently processed may be judged to have only small distracting effects, whereas stimuli that give rise to a feeling of disfluency may be judged to be more distracting. Processing fluency can be manipulated in many different ways, but one of the best established manipulations of processing fluency is repetition (e.g.^22^). Repeatedly presented stimuli are processed more fluently than new stimuli. Just as the direct-access account, the processing-fluency account implies metacognitive judgments of distraction to become less negative with each repetition of the distractor. It is typically assumed that the processing-fluency heuristic is used to arrive at metacognitive judgments because processing fluency has some ecological validity in predicting the to-be-judged dimension in natural environments^23^. It is likely that there are many conditions in which stimuli that are processed less fluently cause more distraction (e.g.^11^). However, in contrast to the direct-access account, the processing-fluency account does not imply that optimal inferences about a stimulus’ distracting potential can always be achieved. In fact, it is possible to specify situations in which the processing-fluency heuristic should lead to metacognitive judgments that are opposed to scientifically established effects of sounds on cognitive performance (see hypotheses below). These situations may not be representative for real-world contingencies between processing fluency and auditory distraction, but from a theoretical perspective, they are particularly interesting because they allow for evaluating the direct-access account against the processing-fluency account.

On the face of it, the literature seems to tentatively support the processing-fluency account over the direct-access account because there are some examples in which metacognitive judgments about the effects of auditory distractors do not directly correspond to the objective sound effects (cf.^24^). Dissociations between metacognitive judgments and objective sound effects are particularly salient in the case of music. For instance, Perham and Sykora^25^ compared the distracting effects of a popular dance track to those of a grindcore metal song. Whereas the dance track contained many changes that are primarily responsible for the distracting effects of music on serial recall^14,26–29^, the individual elements of the grindcore metal song were blended into a “cacophony of sounds” (see^25^, p. 551). As a result, the grindcore metal song objectively caused less distraction than the dance track, but global retrospective metacognitive judgments of distraction did not differ between these two types of sounds. Bell et al.^30^ found that participants who indicated that they liked Mozart’s sonata K. 448 more than other participants gave less negative global retrospective metacognitive judgments about the distracting effects of the music on their performance when in fact the objective sound effect was unrelated to liking. These findings suggest that people do not have direct access to the degree to which music disrupts their serial-recall performance. However, when it comes to testing the processing-fluency account against the direct-access account, both of these studies have important limitations. None of these studies included a direct manipulation of processing fluency. Furthermore, metacognition was only assessed via global retrospective metacognitive judgments, whereas stimulus-specific prospective metacognitive judgments were not assessed. In the study of Perham and Sykora^25^, the two musical pieces that were compared differed greatly in their acoustic properties and not only, if at all, in processing fluency. In the study of Bell et al.^30^, the association between liking and metacognitive judgments of distraction was only correlational; therefore, no causal conclusions could be drawn. A direct test of the competing accounts thus has yet to be performed.

The aim of the present study was to manipulate the processing fluency of music while holding the acoustic complexity of the music constant across conditions. To manipulate processing fluency, the audio files of simple piano melodies or segments of Mozart’s sonata K. 448 were either left in their original forward direction or reversed so as to play the music backward. Reversing the sound files decreases the perceived processing fluency of the music while preserving its acoustic complexity (cf.^31^) (see the “Materials” section of Experiment 1 for a more detailed description of the effects of reversing sound files). Distracting effects of irrelevant sounds on cognitive performance are largely determined by acoustic complexity^13^. This implies that it makes essentially no difference for the objective sound effects on serial-recall performance whether sound is played forward or backward; this has been demonstrated for words, sentences and piano melodies^31–34^. Specifically, Röer et al.^31^ have shown that piano melodies disrupt serial recall to the same degree irrespective of whether they are played forward or backward. The same piano melodies were used in the present Experiment 1.

Comparing metacognitive judgments about the effects of forward and backward music thus provides ideal conditions for testing the direct-access account against the processing-fluency account. The direct-access account implies that people should have access to the processes by which forward music disrupts serial recall just as much as backward music. The processing-fluency account, by contrast, implies that there should be a dissociation between metacognitive judgments and objective sound effects. Given that people are used to the sound of music played in forward direction but not to the sound of music played in backward direction, forward music should be perceived as being easier to process than backward music. The processing-fluency account thus allows predicting a metacognitive illusion in the sense that people incorrectly judge forward music to be less distracting than backward music.

## Experiment 1

### Methods

#### Participants

All participants were recruited on campus at Heinrich Heine University Düsseldorf. We aimed at collecting about 100 valid data sets and stopped data collection at the end of the week in which this criterion was reached. Up to nine participants were tested simultaneously. They were seated in separate cubicles with sound-absorbing walls. Throughout the experiment, participants wore headphones with high-insulation hearing protection covers (beyerdynamic DT-150) plugged directly into Apple iMac computers controlling the experiment. Participants received a small monetary compensation or course credit for participating. Only students from disciplines other than Psychology and non-students were included in the main analysis reported below because it was a priori unclear whether domain-specific knowledge acquired in Psychology lectures would have an effect on the results. The data sets of 39 Psychology students were thus excluded from the analysis. However, we provide a full analysis of the Psychology students’ data at the project’s OSF page (see Data availability statement) showing that the results of the Psychology students largely replicated those of the other participants, even though the metacognitive illusion in the Psychology students’ stimulus-specific prospective metacognitive judgments was numerically less pronounced in that, on average, they judged forward melodies to be slightly distracting. The remaining sample consisted of 122 participants (83 women, 39 men) with a mean age of 22 (*SD* = 4) years. All but three participants were students from various disciplines. Participants were alternately assigned to either the prospective-judgments group (*n* = 62) or the control group (*n* = 60). A sensitivity analysis^35^ showed that, given a sample size of *N* = 122 and α = 0.05, a main effect of the direction of the piano melodies on the objective sound effect of the size η_p_^2^ = 0.10 could be detected with a statistical power of 1 − β = 0.95.

#### Ethics statement

In both experiments reported here, informed consent was obtained from all participants. Approval was received from the ethics committee of the Faculty of Mathematics and Natural Sciences at Heinrich Heine University Düsseldorf for a series of experiments to which the present experiments belong. Both experiments were conducted in accordance with the Declaration of Helsinki.

#### Materials

The piano melodies were eight simple piano melodies in C major that have been used in previous research^9,31,36^. The auditory distractor sequences lasted 8 s and were played binaurally at 65 dB(A). Depending on the condition, the piano melodies were played in their original forward direction or their sound files were reversed so as to play the melodies backward. Reversing the sound file fundamentally alters the characteristics of the piano melody, changing its temporal progression, transforming the melodic contour, inverting the pitch sequences, and swapping the attack and decay phases of individual notes. Thus, while maintaining the overall acoustic complexity of the sound sequence, these alterations change global and local features of the piano melody, as a result of which the backward melody should be perceived as substantially less fluent by the listener because it runs counter to conventional auditory processing patterns. To check whether the manipulation of processing fluency was successful, we asked participants in a separate norming study (*N* = 28, none of whom were Psychology students or had participated in Experiment 1) to rate the processing fluency of the piano melodies. The piano melodies were played to them one by one. As an indicator of the perceived processing fluency, participants rated how difficult or easy it was to listen to these melodies on a scale ranging from − 100 (very difficult) to + 100 (very easy). This single-item processing-fluency scale was used because its validity is supported by findings showing that a single-item scale ranging from difficult to easy reflects various processing-fluency manipulations such as typicality, symmetry, repetition, contrast and pronounceability just as sensitively as a multiple-item scale^37^. With melodies as the units of analysis, the backward melodies (*M* = 26, *SD* = 4) were rated to be less easy to process than the forward melodies (*M* = 75, *SD* = 4), *F*(1, 14) = 592.54, *p* < 0.001, η_p_^2^ = 0.98, supporting the validity of the processing-fluency manipulation.

#### Procedure

**Figure 1:**
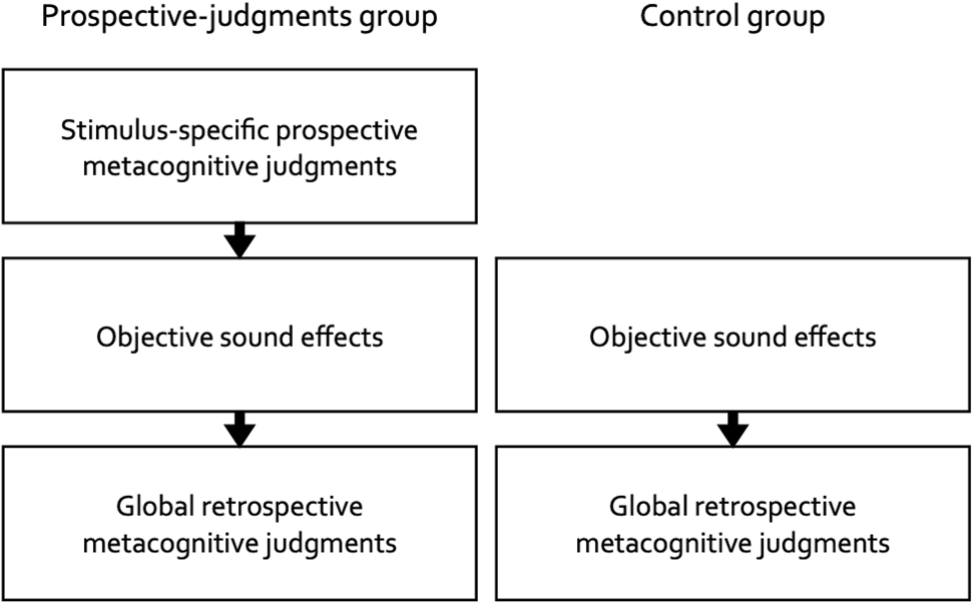
Schematic illustration of the experiment. The experiment consisted of three phases in the prospective-judgments group and of two phases in the control group. Only participants in the prospective-judgments group made stimulus-specific prospective metacognitive judgments about the effects of 16 different piano melodies (eight forward, eight backward) at the beginning of the experiment. All participants performed 29 serial-recall trials (five training trials, then eight quiet control trials, eight distractor trials with forward melodies and eight distractor trials with backward melodies in a different random order for each participant) to measure the objective sound effects on serial recall. At the end of the experiment, all participants made two global retrospective metacognitive judgments about the effects of the two types of piano melodies (forward, backward).

The procedure is illustrated in Fig. 1. Participants in the prospective-judgments group provided stimulus-specific prospective metacognitive judgments about the effects of the piano melodies in a prospective-judgments task before performing the serial-recall task. Participants in the control group started directly with the serial-recall task. This design allows exploring whether the act of providing metacognitive judgments about the effects of the piano melodies on performance would have downstream effects on performance (parallel to similar designs in research on metacognitive judgments of learning; e.g.^4,7,38^) which would be the case, for example, if participants engaged in compensatory efforts to combat distraction after they had reflected on the distracting potential of the sounds (but see^9,10^).

**Figure 2:**
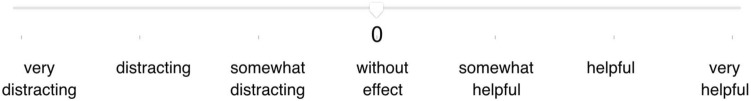
The metacognition scale. Participants judged how distracting or helpful they thought the sound would be (stimulus-specific prospective metacognitive judgment) or had been (global retrospective metacognitive judgment) for their task performance in comparison to quiet. The scale ranged from − 100 (very distracting) to + 100 (very helpful). The figure shows English translations of the German labels with which the scale was presented to the German-speaking participants.

*Stimulus-specific prospective metacognitive judgments* were collected in the prospective-judgments group only. Participants were asked to imagine having to perform a serial-recall task that was described to them. In an example trial, eight different digits appeared at the center of the screen. The digits were shown, one after another, for one second each. The digits were selected at random from the set {1, 2 … 9} without replacement. The randomization process was completely unrestricted, allowing for any combination of digits to randomly occur. Participants were instructed to memorize the order of the digits. Immediately after the last digit had been presented, eight question marks appeared on the screen. The participants were instructed to recall the digits in their correct order. The digits successively replaced the question marks as they were typed into the number pad of the computer’s keyboard. Participants could not correct their responses or skip a digit. After the example trial had been completed, participants received the instructions for the prospective-judgments task. They were informed that various sounds would be played to them over the headphones. They were asked to imagine hearing these sounds while performing the memorization task of which they had just seen an example trial. In each trial of the prospective-judgments task, a button labeled “Play the sound” was shown at the center of the screen. Upon clicking this button, one of the piano melodies was played for eight seconds. Just as in our previous experiment on the metacognition of auditory distraction^11^, the participants indicated on a scale ranging from − 100 (very distracting) to + 100 (very helpful) how distracting or helpful they thought the sound would be for their task performance relative to quiet (see Fig. 2). The judgments about the effects of 16 piano melodies (eight forward, eight backward) were made in an order that was randomized at an individual level.

*Objective sound effects* were assessed after the prospective-judgments task (prospective-judgments group) or at the beginning of the experiment (control group). Participants had to perform a serial-recall task that was described to them. In each trial, eight different digits appeared at the center of the screen. The digits were shown, one after another, for 1 s each. Participants were instructed to memorize the order of the digits without speaking the digits out loud. They were instructed that the sounds they would occasionally hear over the headphones were completely irrelevant to the task. They were asked to ignore everything they heard and to fully concentrate on the digits. Pressing the space bar started the presentation of the to-be-recalled digits. Immediately after the last digit had been presented, participants had to recall the digits in their correct order by typing them into the number pad of the computer keyboard, thereby successively replacing eight question marks on the screen. Participants could not correct their responses or skip a digit. If they did not remember a digit, they were instructed to guess. The serial-recall task started with five training trials in which the digits were presented in quiet. The 24 experimental trials (eight quiet control trials, eight distractor trials with forward melodies, eight distractor trials with backward melodies) followed in an order that was randomized at an individual level. In each of the distractor trials, a different piano melody was played while the digits were presented.

*Global retrospective metacognitive judgments* were collected in both groups immediately after the serial-recall task had been completed. Participants were informed about the type of sound whose effect on the task they had to evaluate (e.g., “In some trials, you have heard a piano melody that was played backward”). They were asked to indicate how distracting or helpful this type of sound had been for their task performance, using the metacognition scale (cf. Fig. 2) ranging from − 100 (very distracting) to + 100 (very helpful). The questions about the effects of forward and backward melodies were asked in a random order.

*Liking judgments* were collected in both groups at the end of the experiment. Participants were informed about the type of sound whose effect on the task they had to evaluate (e.g., “In some trials, you have heard a piano melody that was played backward”). They were asked to indicate how bad or good they found this type of sound, using a liking scale ranging from − 100 (very bad) to + 100 (very good). The questions about forward and backward melodies were asked in a random order.

### Results

#### Stimulus-specific prospective metacognitive judgments

**Figure 3:**
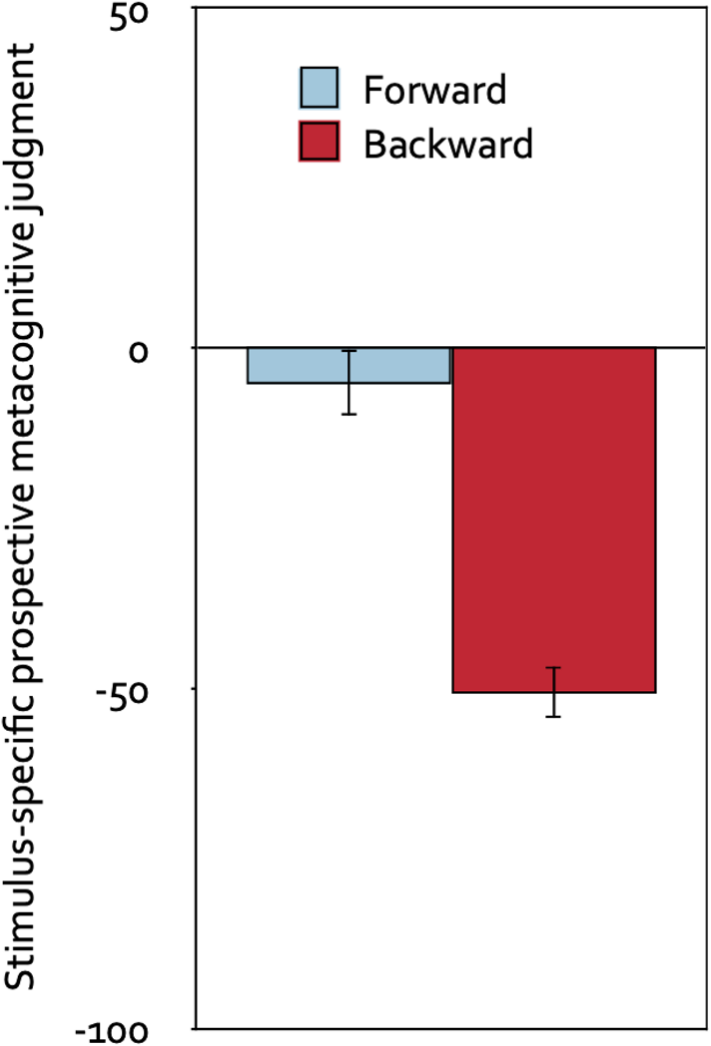
Stimulus-specific prospective metacognitive judgments. Stimulus-specific prospective metacognitive judgments on a scale ranging from − 100 (very distracting) to + 100 (very helpful) as a function of the direction of the piano melodies (forward, backward). The error bars represent the standard errors of the means.

An analysis of variance with direction of the piano melodies (forward, backward) as the repeated-measures factor and stimulus-specific prospective metacognitive judgment as the dependent variable showed that participants in the prospective-judgments group judged the backward melodies to be more distracting than the forward melodies, *F*(1, 61) = 102.00, *p* < 0.001, η_p_^2^ = 0.63. Figure 3 displays the stimulus-specific prospective metacognitive judgment as a function of the direction of the piano melodies. Compared to the neutral midpoint of the scale, the forward melodies were judged to be on average neither helpful nor distracting, *t*(61) =  − 1.09, *p* = 0.282, η_p_^2^ = 0.02, whereas the backward melodies were judged to be distracting, *t*(61) =  − 13.78, *p* < 0.001, η_p_^2^ = 0.76.

#### Objective sound effects

Measures of the objective sound effects on performance were obtained for each participant by subtracting the mean number of digits per trial recalled in the quiet control condition from the mean number of digits per trial recalled in the forward-melody condition and from the mean number of digits per trial recalled in the backward-melody condition. The serial-recall results that formed the basis for calculating the objective sound effects are reported in Table 1.

**Table 1:** The mean number of digits correctly recalled out of the eight digits presented in each trial as a function of the sound condition (quiet control, forward music, backward music) in the serial-recall tasks of Experiments 1 and 2, collapsed over the group factor (prospective-judgments group, control group).

	Quiet control	Forward music	Backward music
Experiment 1	5.38 (0.13)	4.90 (0.14)	4.86 (0.14)
Experiment 2	5.46 (0.13)	4.90 (0.13)	5.05 (0.13)

A strict serial-recall criterion was applied in that only digits recalled at the correct serial positions were scored as correct. Values in parentheses represent the standard errors of the means.

**Figure 4:**
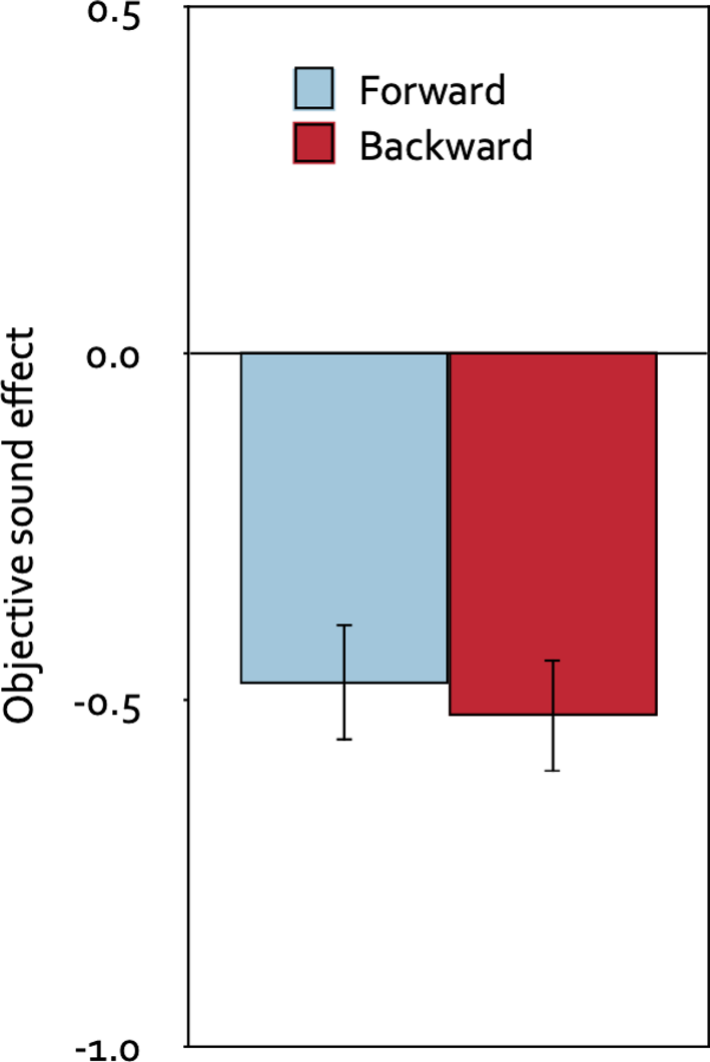
Objective sound effects. Objective sound effects as a function of the direction of the piano melodies (forward, backward) in terms of the difference in the mean number of digits that were correctly recalled in the distractor conditions relative to the quiet control condition per trial, collapsed over the group factor (prospective-judgments group, control group). Negative values indicate a distracting effect of the sounds on serial-recall performance, whereas positive values would indicate a helpful effect. The error bars represent the standard errors of the means.

A 2 × 2 mixed analysis of variance with direction of the piano melodies (forward, backward) as the repeated-measures factor, group (prospective-judgments group, control group) as the between-subjects factor and distraction (the difference between each of the distractor conditions and the quiet control condition in the mean number of digits per trial that were correctly recalled in the serial-recall task) as the dependent variable showed that, objectively, forward melodies disrupted serial recall just as much as backward melodies, *F*(1, 120) = 0.46, *p* = 0.498, η_p_^2^ < 0.01. Whether or not participants were asked to provide stimulus-specific prospective metacognitive judgments about the effects of the piano melodies prior to the serial-recall task had no effect on the overall level of distraction, *F*(1, 120) = 0.22, *p* = 0.644, η_p_^2^ < 0.01, and did not interact with the direction of the piano melodies, *F*(1, 120) = 0.19, *p* = 0.667, η_p_^2^ < 0.01. Therefore, Fig. 4 displays distraction in serial recall as a function of the direction of the piano melodies collapsed over the group factor. Relative to the quiet control condition, participants were distracted by both the forward melodies, *t*(121) =  − 5.59, *p* < 0.001, η_p_^2^ = 0.21, and the backward melodies, *t*(121) =  − 6.51, *p* < 0.001, η_p_^2^ = 0.26.

#### Global retrospective metacognitive judgments

**Figure 5:**
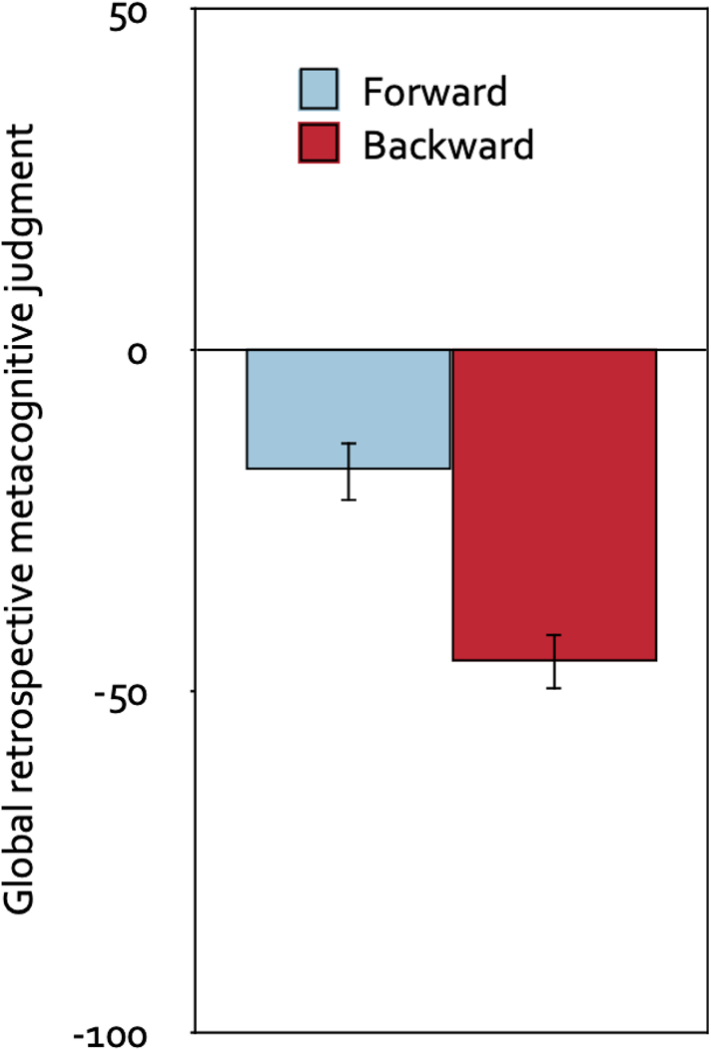
Global retrospective metacognitive judgments. Global retrospective metacognitive judgments on a scale ranging from − 100 (very distracting) to + 100 (very helpful) as a function of the direction of the piano melodies (forward, backward), collapsed over the group factor (prospective-judgments group, control group). The error bars represent the standard errors of the means.

A 2 × 2 mixed analysis of variance with direction of the piano melodies (forward, backward) as the repeated-measures factor, group (prospective-judgments group, control group) as the between-subjects factor and global retrospective metacognitive judgment as the dependent variable showed that participants judged that the backward melodies had been more distracting than the forward melodies, *F*(1, 120) = 37.82, *p* < 0.001, η_p_^2^ = 0.24. Whether or not participants were asked to provide stimulus-specific prospective metacognitive judgments about the effects of the piano melodies prior to the serial-recall task had no effect on the global retrospective metacognitive judgments, *F*(1, 120) = 0.99, *p* = 0.321, η_p_^2^ = 0.01, and did not interact with the direction of the piano melodies, *F*(1, 120) = 2.37, *p* = 0.126, η_p_^2^ = 0.02. Therefore, Fig. 5 displays the global retrospective metacognitive judgment as a function of the direction of the piano melodies collapsed over the group factor. Compared to the neutral midpoint of the scale, both the forward melodies, *t*(121) =  − 4.17, *p* < 0.001, η_p_^2^ = 0.13, and the backward melodies, *t*(121) =  − 11.23, *p* < 0.001, η_p_^2^ = 0.51, were judged to have been distracting.

#### Liking judgments

Participants indicated that they had liked the forward melodies better than the backward melodies, *F*(1, 120) = 116.39, *p* < 0.001, η_p_^2^ = 0.49. Compared to the neutral midpoint of the scale, forward melodies were judged positively (*M* = 26, *SE* = 4), *t*(121) = 7.10, *p* < 0.001, η_p_^2^ = 0.29, whereas backward melodies were judged negatively, (*M* =  − 31, *SE* = 4), *t*(121) =  − 7.19, *p* < 0.001, η_p_^2^ = 0.30. Whether or not participants were asked to provide stimulus-specific prospective metacognitive judgments about the effects of the piano melodies prior to the serial-recall task had no effect on the liking judgments overall, *F*(1, 120) = 0.89, *p* = 0.346, η_p_^2^ = 0.01. However, the difference between forward and backward melodies was more pronounced for those participants who had listened to the melodies to make stimulus-specific prospective metacognitive judgments (*M* = 30, *SE* = 5, for forward melodies; *M* =  − 41, *SE* = 6, for backward melodies) than for those participants who had heard the melodies only when they were trying to ignore them during the serial-recall task (*M* = 22, *SE* = 6, for forward melodies; *M* =  − 21, *SE* = 7, for backward melodies), *F*(1, 120) = 6.81, *p* = 0.010, η_p_^2^ = 0.05.

### Discussion

Replicating previous results^31,39^, the objective effect of the piano melodies on serial-recall performance—as reflected in the difference in serial-recall performance relative to the quiet control condition—was independent of the direction of the piano melodies. Whereas the complexity of the stimulus material and thus the distracting potential of the piano melodies was preserved when the sound files were played backward, subjective ratings of processing fluency indicated that, as anticipated, the backward melodies were perceived as being less easy to process than the forward melodies. The main purpose of Experiment 1 was to test whether the metacognitive judgments would correctly reflect the absence of an effect of the direction of the piano melodies on the objective sound effect as implied by the direct-access account or whether the metacognitive judgments would be affected by the perceived difference in processing fluency. The results favor the processing-fluency account over the direct-access account. The stimulus-specific prospective metacognitive judgments were strongly affected by the direction of the piano melodies. Whereas the backward melodies were predicted to be distracting, forward melodies were on average not predicted to be distracting. A difference between backward and forward melodies was preserved in the global retrospective metacognitive judgments that were made after participants had just experienced the distracting effect of the piano melodies on serial recall first-hand. Even though in retrospect participants correctly judged the forward melodies to have been distracting, there was still a significant effect of the direction of the piano melodies in that participants judged the backward melodies to have been more distracting than the forward melodies. This finding suggests that the direct experience of performing the task in the face of distraction may mitigate, but does not fully eliminate, the metacognitive illusion.

## Experiment 2

Given that it is problematic to draw conclusions based on the results of a single study^40^, the main purpose of Experiment 2 was to serve as a close replication of Experiment 1. The basic procedure was identical to that of Experiment 1 with the main exception being that 8-s segments of Mozart’s sonata K. 448 (cf.^30^) were used as the distractor material instead of the piano melodies that were used in Experiment 1. The processing-fluency account implies that the effect of the direction of the music should not depend on the specific music used. If the processing-fluency account is valid, the results of Experiment 1 should be replicated in Experiment 2.

### Methods

#### Participants

All participants were recruited on campus at Heinrich Heine University Düsseldorf. We aimed at collecting about 100 valid data sets and stopped data collection at the end of the week in which this criterion was reached. Up to five participants were tested simultaneously. They were seated in separate cubicles with sound-absorbing walls. Throughout the experiment, participants wore headphones with high-insulation hearing protection covers (beyerdynamic DT-150) plugged directly into Apple iMac computers controlling the experiment. Only students from disciplines other than Psychology and non-students were included in the main analysis. The data sets of 75 Psychology students were excluded from the analysis. A full analysis of the Psychology students’ data is reported at the project’s OSF page (see Data availability statement) showing that the results of the Psychology students largely replicated those of the other participants, with the exception that the metacognitive illusion in the stimulus-specific prospective metacognitive judgments was numerically less pronounced than that of the other participants because Psychology students judged forward segments of Mozart’s sonata K. 448 to be slightly distracting. The remaining sample consisted of 101 participants (65 women, 36 men) with a mean age of 23 (*SD* = 4) years. All participants but one were students from various disciplines. Participants were alternately assigned to either the prospective-judgments group (*n* = 51) or the control group (*n* = 50). A sensitivity analysis^35^ showed that, given a sample size of *N* = 101 and α = 0.05, a main effect of the direction of the segments of Mozart’s sonata K. 448 on the objective sound effect of the size η_p_^2^ = 0.12 could be detected with a statistical power of 1 − β = 0.95.

#### Materials and procedure

Materials and procedure were identical to those of Experiment 1 with the following exception: Instead of the eight piano melodies that were specifically composed for research purposes, we used eight different 8-s segments of Mozart’s sonata K. 448. Depending on the condition, the segments of Mozart’s sonata K. 448 were left in their original forward direction or the sound files were reversed so as to play the segments backward. Note that if participants were familiar with Mozart’s sonata K. 448, this might amplify the differences in processing fluency between the forward and backward segments as familiarity is assumed to contribute to processing fluency^41,42^. To check whether the manipulation of processing fluency was successful, participants in a separate norming study (*N* = 27, none of whom were Psychology students or had participated in Experiments 1 or 2) were asked to rate how difficult or easy it was to process the 8-s segments of Mozart’s sonata K. 448 on the single-item processing-fluency scale that has been used in Experiment 1 (cf.^37^). With segments of Mozart’s sonata K. 448 as the units of analysis, the backward segments (*M* = 13, *SD* = 7) were rated to be less easy to process than the forward segments (*M* = 74, *SD* = 9), *F*(1, 14) = 224.31, *p* < 0.001, η_p_^2^ = 0.94, supporting the validity of the processing-fluency manipulation.

### Results

#### Stimulus-specific prospective metacognitive judgments

**Figure 6:**
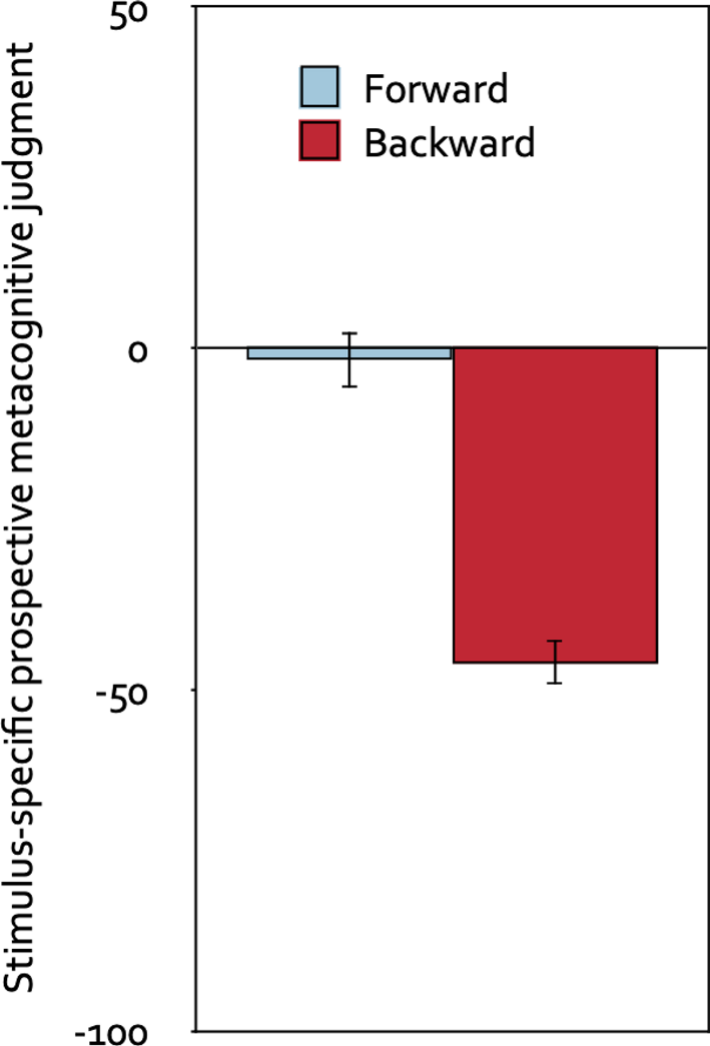
Stimulus-specific prospective metacognitive judgments. Stimulus-specific prospective metacognitive judgments on a scale ranging from − 100 (very distracting) to + 100 (very helpful) as a function of the direction of the segments of Mozart’s sonata K. 448 (forward, backward). The error bars represent the standard errors of the means.

An analysis of variance with direction of the segments of Mozart’s sonata K. 448 (forward, backward) as the repeated-measures factor and stimulus-specific prospective metacognitive judgment as the dependent variable showed that participants in the prospective-judgments group judged the backward segments to be more distracting than the forward segments, *F*(1, 50) = 133.53, *p* < 0.001, η_p_^2^ = 0.73. Figure 6 displays the stimulus-specific prospective metacognitive judgment as a function of the direction of the segments. Compared to the neutral midpoint of the scale, forward segments of Mozart’s sonata K. 448 were judged to be on average neither helpful nor distracting, *t*(50) =  − 0.47, *p* = 0.641, η_p_^2^ < 0.01, whereas backward segments were judged to be distracting, *t*(50) =  − 14.75, *p* < 0.001, η_p_^2^ = 0.81.

#### Objective sound effects

**Figure 7:**
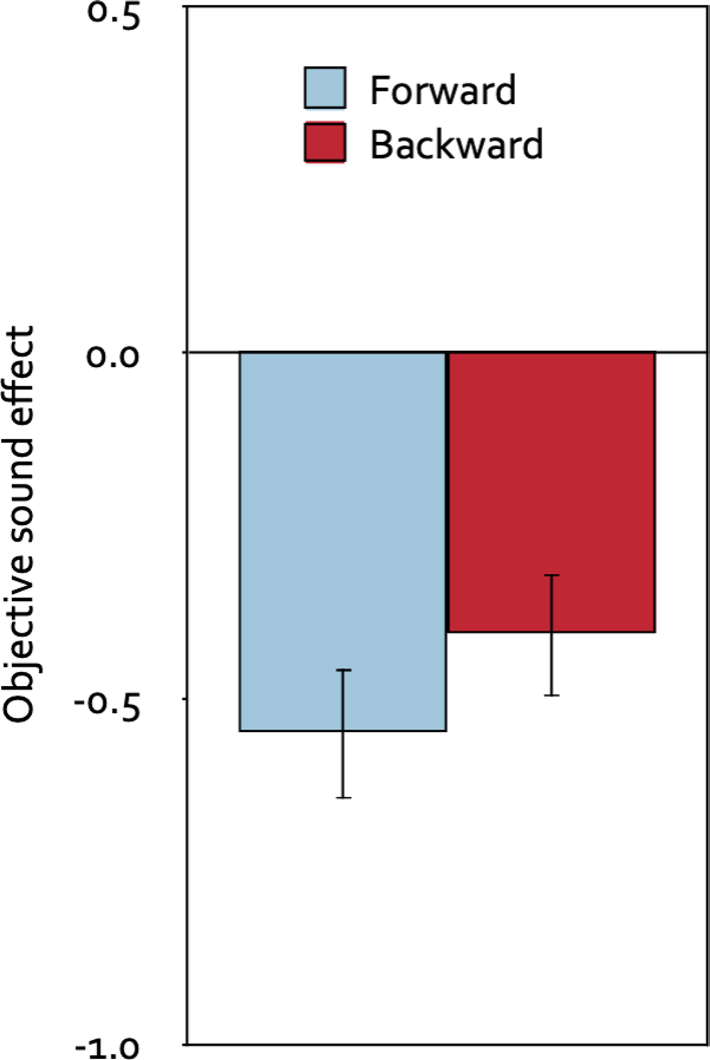
Objective sound effects. Objective sound effects as a function of the direction of the segments of Mozart’s sonata K. 448 (forward, backward) in terms of the difference in the mean number of digits that were correctly recalled in the distractor conditions relative to the quiet control condition per trial, collapsed over the group factor (prospective-judgments group, control group). Negative values indicate a distracting effect of the sounds on serial-recall performance, whereas positive values would indicate a helpful effect. The error bars represent the standard errors of the means.

Measures of the objective sound effects on performance were obtained for each participant by subtracting the mean number of digits per trial recalled in the quiet control condition from the mean number of digits per trial recalled in the forward-segment condition and from the mean number of digits per trial recalled in the backward-segment condition. The serial-recall results that formed the basis for calculating the objective sound effects are reported in Table 1. A 2 × 2 mixed analysis of variance with direction of the segments of Mozart’s sonata K. 448 (forward, backward) as the repeated-measures factor, group (prospective-judgments group, control group) as the between-subjects factor and distraction (the difference between each of the distractor conditions and the quiet control condition in the mean number of digits per trial that were correctly recalled in the serial-recall task) as the dependent variable showed that, objectively, forward segments of Mozart’s sonata K. 448 disrupted serial recall just as much as backward segments, *F*(1, 99) = 1.99, *p* = 0.161, η_p_^2^ = 0.02. Whether or not participants were asked to provide stimulus-specific prospective metacognitive judgments about the effects of the segments of Mozart’s sonata K. 448 prior to the serial-recall task had no effect on the overall level of distraction, *F*(1, 99) < 0.01, *p* = 0.998, η_p_^2^ < 0.01, and did not interact with the direction of the segments, *F*(1, 99) = 0.21, *p* = 0.648, η_p_^2^ < 0.01. Therefore, Fig. 7 displays distraction in serial recall as a function of the direction of the segments of Mozart’s sonata K. 448 collapsed over the group factor. Relative to the quiet control condition, participants were distracted by both the forward segments, *t*(100) =  − 5.85, *p* < 0.001, η_p_^2^ = 0.26, and the backward segments, *t*(100) =  − 4.71, *p* < 0.001, η_p_^2^ = 0.18.

#### Global retrospective metacognitive judgments

**Figure 8:**
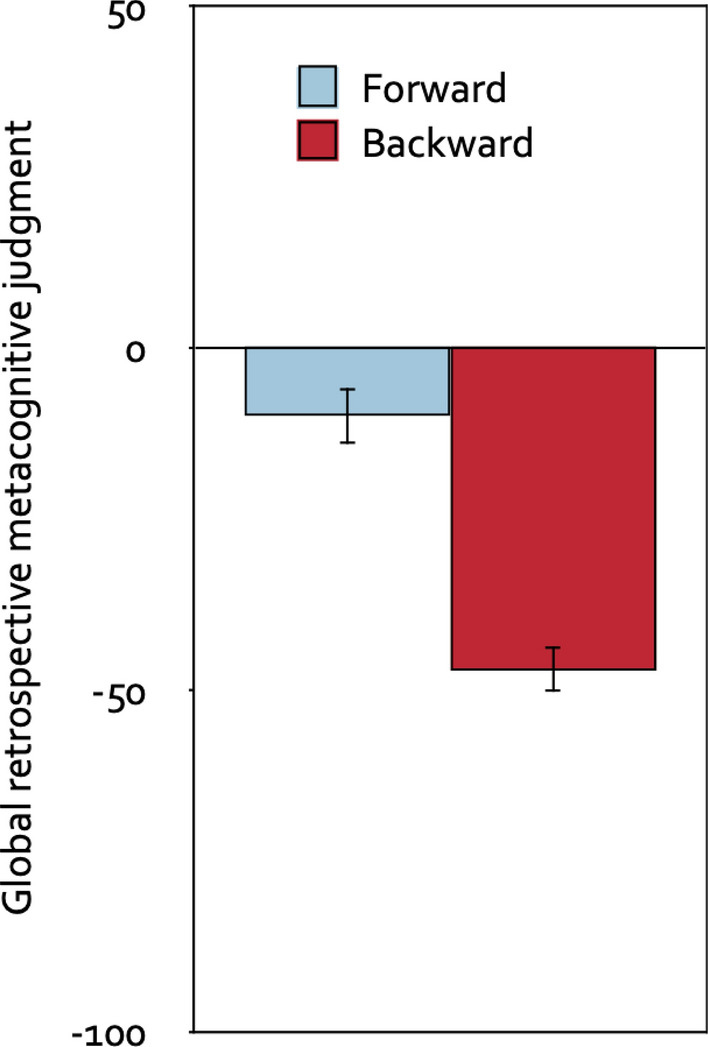
Global retrospective metacognitive judgments. Global retrospective metacognitive judgments on a scale ranging from − 100 (very distracting) to + 100 (very helpful) as a function of the direction of the segments of Mozart’s sonata K. 448 (forward, backward), collapsed over the group factor (prospective-judgments group, control group). The error bars represent the standard errors of the means.

A 2 × 2 mixed analysis of variance with direction of the segments of Mozart’s sonata K. 448 (forward, backward) as the repeated-measures factor, group (prospective-judgments group, control group) as the between-subjects factor and global retrospective metacognitive judgment as the dependent variable showed that participants judged the backward segments to have been more distracting than the forward segments, *F*(1, 99) = 82.48, *p* < 0.001, η_p_^2^ = 0.45. Whether or not participants were asked to provide stimulus-specific prospective metacognitive judgments about the effects of the segments of Mozart’s sonata K. 448 prior to the serial-recall task had no effect on the global retrospective metacognitive judgments, *F*(1, 99) = 0.52, *p* = 0.472, η_p_^2^ = 0.01, and did not interact with the direction of the segments, *F*(1, 99) = 1.43, *p* = 0.235, η_p_^2^ = 0.01. Therefore, Fig. 8 displays the global retrospective metacognitive judgment as a function of the direction of the segments of Mozart’s sonata K. 448 collapsed over the group factor. Compared to the neutral midpoint of the scale, both the forward segments, *t*(100) =  − 2.44, *p* = 0.016, η_p_^2^ = 0.06, and the backward segments, *t*(100) =  − 14.14, *p* < 0.001, η_p_^2^ = 0.67, were judged to have been distracting.

#### Liking judgments

Participants indicated that they had liked the forward segments of Mozart’s sonata K. 448 better than the backward segments, *F*(1, 99) = 160.70, *p* < 0.001, η_p_^2^ = 0.62. Compared to the neutral midpoint of the scale, forward segments were judged positively (*M* = 46, *SE* = 4), *t*(100) = 12.32, *p* < 0.001, η_p_^2^ = 0.60, whereas backward segments were judged negatively, (*M* =  − 25, *SE* = 4), *t*(100) =  − 5.67, *p* < 0.001, η_p_^2^ = 0.24. Participants who had listened to the segments of Mozart’s sonata K. 448 to make stimulus-specific prospective metacognitive judgments on average liked the segments less than participants who had heard the segments only when they were trying to ignore them during the serial-recall task, *F*(1, 99) = 7.44, *p* = 0.008, η_p_^2^ = 0.07. The difference between forward and backward segments of Mozart’s sonata K. 448 was more pronounced for participants in the prospective-judgments group (*M* = 46, *SE* = 5, for forward segments; *M* =  − 41, *SE* = 5, for backward segments) than for participants in the control group (*M* = 45, *SE* = 6, for forward segments; *M* =  − 10, *SE* = 7, for backward segments), *F*(1, 99) = 7.96, *p* = 0.006, η_p_^2^ = 0.07.

### Discussion

The pattern of statistical significance obtained in Experiment 1 was replicated in Experiment 2. This successful replication indicates that the metacognitive illusion in the judgments of distraction is robust and does not depend on the specific type of music. Note that if participants were familiar with Mozart’s sonata K. 448, this could have contributed to the differences in processing fluency between the forward and backward segments of the sonata^41,42^. However, the fact that the results are so strikingly consistent between Experiments 1 and 2 suggests that these effects are primarily due to the disfluency resulting from reversing the sound files, rather than a specific familiarity with Mozart’s sonata K. 448.

## General discussion

The purpose of the present experiments was to examine how people evaluate the distracting effects of sounds on performance. Two accounts were tested against each other. According to the direct-access account, people have direct access to the processes that underlie the distracting effects of the sounds. Specifically, people may have access to the degree to which different types of stimuli attract their focus of attention^18,19^. According to the processing-fluency account, people heuristically judge the distracting effects of the sounds by relying on processing fluency. To tease these two possibilities apart, we focused on a case in which the two accounts imply different predictions. Previous studies^31,39^ have demonstrated that backward music is as distracting as forward music, presumably because the sound files contain the same amount of information that is to be processed regardless of whether the sound files are reversed or not. Replicating these earlier findings, both experiments presented here confirm that the distracting effects of music on serial-recall performance do not depend on the direction of the music. The direct-access account implies that, when being asked to judge the distracting effects of concrete examples of music, people should have access to the fact that backward and forward music is equally distracting. However, backward music should give rise to a reduced feeling of fluency compared to forward music. Providing direct empirical support for this prediction, subjective processing-fluency ratings differed as a function of the direction of the music. The backward piano melodies or segments of Mozart’s sonata K. 448 were rated to be less easy to process than the forward melodies or sonata segments. As implied by the processing-fluency account, participants may be misled when they use the processing-fluency heuristic as the basis for their metacognitive judgments. Specifically, the less fluent processing of backward music should lead participants to judge the piano melodies or segments of Mozart’s sonata K. 448 as being more distracting than the forward melodies or sonata segments. The results favor the processing-fluency account over the direct-access account. Participants prospectively judged backward music to be more distracting than forward music. Whereas the backward piano melodies or segments of Mozart’s sonata K. 448 were judged to be disruptive to serial-recall performance relative to quiet, the forward melodies or sonata segments were predicted not to be distracting. This provides evidence of a metacognitive illusion due to the use of the processing-fluency heuristic. This conclusion is in line with the conclusions reached for metacognitive judgments in other domains such as the judgments of learning: People lack direct introspective access to their own cognitive processes^43^ and have to use the fluency heuristic instead. Specifically, to-be-learned material that gives rise to a feeling of fluency is often judged to be easier to learn and to remember than to-be-learned material that promotes a feeling of disfluency^44^.

To test the robustness of the findings, Experiment 2 served as a close replication of Experiment 1, using segments of Mozart’s sonata K. 448 instead of the simple piano melodies that had been created for research purposes^31^. Against the background of the discussion about the robustness of psychological findings^40^, a rigorous test of reproducibility is an essential step for establishing novel findings^45,46^. As a close replication with different stimuli, Experiment 2 goes beyond the mere demonstration of reproducibility by showing that the metacognitive illusion observed in Experiment 1 does not depend on peculiarities of the stimulus material. Now that the metacognitive illusion has been established to be robustly present with different types of music, future studies can extend these findings to other categories of sounds. From the processing-fluency account supported by the data obtained in the present experiments, it is possible to derive the prediction that the metacognitive illusion should not be limited to the types of music used here or to music in general but should extend to all situations in which the perceived processing fluency and the objective effects of task-irrelevant sounds are in conflict with each other. The theoretical framework thus is productive in that it allows to derive novel hypotheses that can guide future research. Apart from testing whether the processing-fluency account generalizes to other categories of sounds, it will be important to examine how metacognitive judgments about the distracting effects of sounds translate into decisions of whether to avoid or to seek out exposure to these sounds. It will also be important to extend the present research by focusing on situations in which the processing-fluency heuristic leads to correct judgments. When interpreting the present findings, it should be kept in mind that we deliberately focused on a case in which relying on the processing-fluency heuristic causes an illusion. This is likely the exception rather than the rule. To illustrate, there has been a close correspondence between the metacognitive judgments and the objective sound effects on performance in a previous study^11^ that focused on stimulus-specific prospective and global retrospective metacognitive judgments about the effects of steady-state, auditory-deviant and changing-state sequences on serial recall. Both the objective sound effects and the metacognitive judgments of distraction were found to become more positive as a function of repetition. Given that repetition is a main determinant of processing fluency (e.g.^22^), these findings can be explained by the heuristic use of processing fluency which, in that case, resulted in correct judgments. Thus, even though relying on processing fluency can be demonstrated to predictably lead to incorrect judgments about the distracting effect of music, this does not necessarily imply that the processing-fluency heuristic lacks ecological validity in everyday life.

After completing the serial-recall task, participants retrospectively judged the forward music to have been slightly distracting relative to quiet, but participants still incorrectly judged that the backward music had been more distracting than the forward music. This finding suggests that the direct experience of performing the task in the face of distraction may mitigate the metacognitive illusion (see^24^ for a consistent finding) presumably because participants were able to use, at least to some extent, cues that became available when they had performed the task in the face of distraction. For example, the participants may have noticed that they were less successful in memorizing the digits when forward and backward music was played than when no music was played. However, evidently these cues could not fully eliminate the metacognitive illusion.

Processing fluency naturally carries a positive affective quality^47,48^. It has been suggested that, in case of judgments in the evaluative domain such as liking judgments, affective responses serve as the proximal cues that are ultimately responsible for the processing-fluency effects^49^. By contrast, in case of metacognitive judgments about cognitive processes such as learning or distraction, the proximal cue that is thought to underlie processing-fluency effects is the experienced ease or difficulty of processing^3,5,11,17,21,50,51^. Against this backdrop, it seems interesting that the liking judgments show a somewhat different pattern than the global retrospective metacognitive judgments, suggesting that the effects of the direction of music on the global retrospective metacognitive judgments do not directly correspond to changes in affect. Nevertheless, future studies could address the potential contribution of the affective quality of the task-irrelevant sounds to the effects of processing fluency on the metacognitive judgments more directly.

To summarize, here we examined how people arrive at metacognitive judgments about the effects of music on cognitive performance. The results provide evidence for a metacognitive illusion. The objective performance measures showed that forward and backward music disrupts serial recall to the same degree, replicating the findings of previous studies^31,39^. However, when participants were asked to make stimulus-specific prospective metacognitive judgments about the effects of forward and backward music they were given a chance to listen to, they incorrectly judged the backward music to be more distracting to serial-recall performance than the more fluently processed forward music; in fact, participants did not ascribe any distracting effect to forward music at all. When participants made global retrospective metacognitive judgments about the effects of music, forward music was judged to be slightly distracting but still much less distracting than backward music. The results thus suggest that people rely on the processing-fluency heuristic to arrive at metacognitive judgments about the effects of music on serial-recall performance and that they have no direct access to the processes underlying auditory distraction. This makes people vulnerable to a metacognitive illusion. Potentially, this metacognitive illusion in the judgments of distraction could result in poor metacognitive judgments about the environmental conditions that promote good learning performance. For instance, people may choose to study in the presence of music they find easy to listen to but which in fact is detrimental to their performance. It thus seems important to educate the public about the factors that cause distraction.

## Data Availability

The data of both experiments and supplementary analyses are available at the project page of the Open Science Framework under https://osf.io/j3r2e/.
